# Supplementary material for: Process evaluation of enhancing primary health care for non-communicable disease management in Malaysia: Uncovering the fidelity & feasibility elements
Source: PLoS One. 2021 Jan 11;16(1):e0245125. doi: 10.1371/journal.pone.0245125 (PMC7799751; doi:10.1371/journal.pone.0245125)
Supplement: S1 Checklist — (PDF) [file pone.0245125.s001.pdf]

TO BE FILLED BY RESEARCHER								
Researcher:								
State:	Selangor	Johor		Type of KK:	2	3	4	
Health Clinic's Name :				Date :	/	/	201	

ENHANCED PRIMARY HEALTHCARE – PROCESS EVALUATION
Intervention Evaluation Form (PE02)

			Page
☐	**Section A**	**: Clinic Demography**<br>A1. How does the LO rate the clinic workload today?<br>A2. Clinic's ICT Facility<br>A3. Strength of Family Health Team (FHT) in the clinic	2
☐	**Section B1**	**: Primary Triage**<br>B101. Primary Triage Counter	3
☐	**Section B2**	**: Registration**<br>B201. Signage and Clinic Floor Plan<br>B202. Fee Schedule<br>B203. Registry to PER-PL102<br>B204. Population Registry (MOVes)	4-7
☐	**Section B3**	**: Secondary Triage**<br>B301. Secondary Triage Counter<br>B302. Risk Stratification Criteria & Standard Operating Protocol<br>B303. Patient Screening Criteria<br>B304. NCD Screening Form<br>B305. NCD Appointment Book<br>B306. Health Education	8-11
☐	**Section B4**	**: Pharmacy**<br>B401. Medication Therapy Adherence Clinic (MTAC) Services in General<br>B402. Mechanism to assess adherence status<br>B403. Mechanism to inform defaulter to care coordinator<br>B404. MALMAS form	12-13
☐	**Section B5**	**: Management Audit**<br>B501. Audit Criteria Document<br>B502. Audit Schedule<br>B503. Customer Satisfaction Survey	14-15
☐	**Section B6**	**: Care Coordinator**<br>B601. Visit Checklist<br>B602. NCD Care Form<br>B603. Mechanism to trace appointments<br>B604. Mechanism to trace defaulters<br>B605. Referral Registry	16-17
☐	**Section B7**	**: Integrated Specialised Services**	18
☐	**Section C**	**: Researcher's Notes**	18
☐	**Section D**	**: Clinic Layout**	19

Note to researchers: Please give more attention on facilitators and barriers to the implementation.

SECTION A (Clinic Demography)

Researchers to do a brief interview for this section with either the clinic's Liaison Officer (LO), Medical Officer in-charge or Family Medicine Specialist.

A1. How does the LO rate the clinic workload today?

Busier than usual | As per usual clinic days | Less busy than usual

A2. Clinic's ICT Facility

**A201**	**Registration** (EPRS, TPC and/or OHCIS)	:	1 **Yes**	2 **No**
**A202**	**Operational** (TPC and/or OHCIS)	:	1 **Yes**	2 **No**
**A203**	**Pharmacy** (PhIS or equivalent)	:	1 **Yes**	2 **No**
**A204**	**Laboratory** (i-LAB or equivalent)	:	1 **Yes**	2 **No**
**A205**	**Queue Management System (QMS)**	:	1 **Yes**	2 **No**
	a) Is QMS deployed for all services?	:	1 **All services**	2 **Selected services**

Comments

Specify:

A3. Strength of Family Health Team (FHT) in the Clinic

A301 How many FHT were created in the clinic? : ________team(s) ☐ Have yet to be created

A302 Composition of staff in a FHT in the clinic

(Please tick below. Circle V if visiting staff, circle CC if care coordinator, circle HE if staff also acts as a health educator)

Staff Category	FHT #1	FHT #2	FHT #3	FHT #4	FHT #5	If in multiple team	not in specific team/zone
a) Family Medicine Specialist	V / CC / HE	V / CC / HE	V / CC / HE	V / CC / HE	V / CC / HE		
b) Medical Officer	V / CC / HE	V / CC / HE	V / CC / HE	V / CC / HE	V / CC / HE		
c) Nurse	V / CC / HE	V / CC / HE	V / CC / HE	V / CC / HE	V / CC / HE		
d)Community Nurse	V / CC / HE	V / CC / HE	V / CC / HE	V / CC / HE	V / CC / HE		
e) Assistant Medical Officer	V / CC / HE	V / CC / HE	V / CC / HE	V / CC / HE	V / CC / HE		
f) Pharmacist	V / CC / HE	V / CC / HE	V / CC / HE	V / CC / HE	V / CC / HE		
g) Nutritionist	V / CC / HE	V / CC / HE	V / CC / HE	V / CC / HE	V / CC / HE		
h) Diabetic Educator	V / CC / HE	V / CC / HE	V / CC / HE	V / CC / HE	V / CC / HE		
i) Physiotherapist	V / CC / HE	V / CC / HE	V / CC / HE	V / CC / HE	V / CC / HE		
j)	V / CC / HE	V / CC / HE	V / CC / HE	V / CC / HE	V / CC / HE		
k)	V / CC / HE	V / CC / HE	V / CC / HE	V / CC / HE	V / CC / HE		

Any issues/problems regarding FHT?
Is no. of FHT = no. of zones?
If there is a staff in multiple teams, why?

SECTION B1 (Primary Triage)

Respondent :	Medical Officer in-charge	Liaison Officer	Person in-charge	Others

B101 Primary Triage Counter

Availability			
Yes	If YES, is it used? Yes / No	**Who manned the counter?** *(staff category)* :	
		**How many personnel?** *(at one point of time)* :	
	**Location**	**Comments** (include Adaptability, if any)	
No	**If NO, why?**		

Researcher's Observation (to also observe the tasks performed at the primary triage counter)

SECTION B2 (Registration)

Respondent :	Medical Officer in-charge	Liaison Officer	Person in-charge	Others

B201 Signage and Clinic Floor Plan

**Availability**		
Yes	**Implemented before or after EnPHC?** :	
	**If implemented before EnPHC, any workflow difference?** :	
	**Facilitators/barriers in implementation?** :	
No	**If NO, why?**	

Researcher's Observation

SECTION B2 (Registration) …continued

B202 Fee Schedule		
**Availability**		
Yes	**Implemented before or after EnPHC?** :	
	**If implemented before EnPHC, any workflow difference?** :	
	**Facilitators/barriers in implementation?** :	
No	**If NO, why?**	
**Researcher's Observation**		

SECTION B2 (Registration) …continued

**B203 Registry to PER-PL102** (clinic attendance book)		
**Availability**		
Yes	**Implemented before or after EnPHC?** :	
	**If implemented before EnPHC, any workflow difference?** :	
	**Facilitators/barriers in implementation?** :	
No	**If NO, why?**	
**Researcher's Observation**		

SECTION B2 (Registration) …continued

**B204 Population Registry (MOVes)**		
**Availability**		
Yes	**If YES, is it used?**<br>Yes No	**Comments** (include Adaptability, if any)
No	**If NO, why?**	
**Researcher's Observation**		

SECTION B3 (Secondary Triage)

Respondent :	Medical Officer in-charge	Liaison Officer	Person in-charge	Others

B301 Secondary Triage Counter

Availability

Yes	**If YES, is it used?** Yes / No	**Who manned the counter?** : **How many personnel?** :
	**Location**	**Comments** (include Adaptability, if any)
No	**If NO, why?**	

Researcher's Observation

B302 Risk Stratification Criteria & Standard Operating Protocol

Availability

Yes	**If YES, is it used?** Yes / No	**Comments** (include Adaptability, if any)
No	**If NO, why?**	

Researcher's Observation

SECTION B3 (Secondary Triage) …continued

**B303 Patient Screening Criteria**		
**Availability**		
Yes	**If YES, is it used?** Yes No	**Comments** (include Adaptability, if any)
No	**If NO, why?**	
**Researcher's Observation**		

**B304 NCD Screening Form**		
**Availability**		
Yes	**If YES, is it used?** Yes No	**Comments** (include Adaptability, if any)
No	**If NO, why?**	
**Researcher's Observation**		

SECTION B3 (Secondary Triage) …continued

**B305 NCD Appointment Book**		
**Availability**		
Yes	**If YES, is it used?** Yes No	**Comments** (include Adaptability, if any)
No	**If NO, why?**	
**Researcher's Observation**		

SECTION B3 (Secondary Triage) …continued

**B306 Health Education**			
**Availability**			
Yes	**Implemented before or after EnPHC?**	:	
	**If implemented before EnPHC, any workflow difference?**	:	
	**Facilitators/barriers in implementation?**	:	
No	**If NO, why?**		
**Researcher's Observation**			

SECTION B4 (Pharmacy)

Respondent :	Medical Officer in-charge	Liaison Officer	Person in-charge	Others

B401 Medication Therapy Adherence Clinic (MTAC) Services in General

Availability		
Yes	Dedicated room? Yes / No	How many pharmacists? : Service implementation : ☐ Separate from FHT ☐ Together with FHT
	Location	
	Implemented before or after EnPHC? :	
	If implemented before EnPHC, any workflow difference? :	
	Facilitators/barriers in implementation? :	
No	If NO, why?	

Researcher's Observation

SECTION B4 (Pharmacy) …continued

B402 Mechanism to assess adherence status

Availability	
Yes	**What is the mechanism?** (include Adaptability, if any)
No	**If NO, why?** (to probe Adaptability, if any)

B403 Mechanism to inform defaulter to care coordinator

Availability	
Yes	**What is the mechanism?** (include Adaptability, if any)
No	**If NO, why?** (to probe Adaptability, if any)

B404 MALMAS form

Availability		
Yes	**If YES, is it used?** Yes No	**Comments** (include Adaptability, if any)
No	**If NO, why?**	

Researcher's Observation

SECTION B5 (Management Audit)

Respondent :	Medical Officer in-charge	Liaison Officer	Person in-charge	Others

B501 Audit Criteria Document

**Availability**		
Yes	**Implemented before or after EnPHC?** :	
	**If implemented before EnPHC, any workflow difference?** :	
	**Facilitators/barriers in implementation?** :	
No	**If NO, why?**	

Researcher's Observation

SECTION B5 (Management Audit) …continued

B502 Audit Schedule

Availability

Yes	**Implemented before or after EnPHC?** :	
	**If implemented before EnPHC, any workflow difference?** :	
	**Facilitators/barriers in implementation?** :	
No	**If NO, why?**	

Researcher's Observation

B503 Customer Satisfaction Survey (Please take note that this EnPHC intervention is different from the Kawanku survey)

Availability

Yes	**If YES, is it used?** Yes / No	**Comments** (include Adaptability, if any)
No	**If NO, why?**	

Researcher's Observation

SECTION B6 (Care Coordinator)

Respondent	:	Medical Officer in-charge	Liaison Officer	Person in-charge	Others

B601 Visit Checklist

Availability		
Yes	**If YES, is it used?** Yes / No	**Comments** (include Adaptability, if any)
No	**If NO, why?**	

Researcher's Observation

B602 NCD Care Form

Availability		
Yes	**If YES, is it used?** Yes / No	**Comments** (include Adaptability, if any)
No	**If NO, why?**	

Researcher's Observation

SECTION B6 (Care Coordinator) …continued

B603 Mechanism to trace appointments

Availability	
Yes	**What is the mechanism?** (include Adaptability, if any)
No	**If NO, why?** (to probe Adaptability, if any)

B604 Mechanism to trace defaulters

Availability	
Yes	**What is the mechanism?** (include Adaptability, if any)
No	**If NO, why?** (to probe Adaptability, if any)

B605 Referral Registry

Availability		
Yes	**If YES, is it used?** Yes / No	**Comments** (include Adaptability, if any)
No	**If NO, why?**	

Researcher's Observation

SECTION B7 (Integrated Specialised Services)

Respondent	:	Medical Officer in-charge	Liaison Officer	Person in-charge	Others

B701 Integrated Specialised Services (ISS)

Availability				
Yes	**If YES, is it used?**		**Who managed this service?** *(staff category)*	:
	Yes	No	**How many personnel?** *(at one point of time)*	:
	**Location**		**Comments** (include Adaptability, if any)	
No	**If NO, why?**			

Researcher's Observation

SECTION C (Researcher's Notes)

SECTION D (Clinic Layout)

- Draw a rough sketch of the clinic floor plan
  - Estimate the room length & width measurement in meters, or the ratio between room length & width.
- Please pay special attention regarding the locations of the Primary Triage, the Registration counter, the Secondary Triage, the Consultation rooms, and the Pharmacy.
  - If the intervention(s) is located in another building, please estimate the distance between the buildings.
